# Novel Analysis of Coronary Angiography in Predicting the Formation of Ventricular Aneurysm in Patients With Acute Myocardial Infarction After Percutaneous Coronary Intervention

**DOI:** 10.3389/fcvm.2022.880289

**Published:** 2022-04-28

**Authors:** Pujiao Yu, Peng Xi, Yu Tang, Jiahong Xu, Yang Liu

**Affiliations:** Department of Cardiology, Shanghai Tongji Hospital, Tongji University School of Medicine, Shanghai, China

**Keywords:** ventricular aneurysm, acute myocardial infarction, percutaneous coronary intervention, coronary angiogram (CAG), prognosis

## Abstract

**Background:**

Ventricular aneurysm (VA) is a serious complication of acute myocardial infarction (AMI), with a very poor prognosis. Early-stage prophylactic treatment is effective in preventing the formation of VAs. However, the existing predictive models for VA formation lack the sensitivity and specificity necessary for evaluating patients with MI. This study aimed to explore the potential use of coronary angiography and establish a more precise prediction model for VA in patients with MI.

**Methods:**

Patients with VA (*n* = 52) admitted to our medical center between June 2020 and July 2021 with previous emergency percutaneous coronary intervention for AMI were retrospectively included in this database study. Controls that matched 4:1 with the VA cases during the same period were enrolled. The baseline characteristics and coronary angiograms of the enrolled individuals were obtained from the electronic medical record system. The curve length of the distance from the main criminal lesion to its ostia (DLO) and distal (DLD) in the coronary artery were measured with ImageJ. Binary logistic regression analysis was used to identify the predictive factors. The model performance was evaluated by receiver operating characteristic curve analysis.

**Results:**

Binary analysis revealed maximum serum cardiac troponin I level (odds ratio [OR] = 1.046, 95% confidence interval [CI] = 1.027–1.066, *P* < 0.001), serum brain natriuretic peptide level (OR = 1.001, 95% CI = 1.000–1.002, *P* = 0.007), left anterior descending artery as the culprit lesion (OR = 5.091, 95% CI = 2.080–12.457, *P* < 0.001), and that single-vessel disease (OR = 1.809, 95% CI = 0.967–3.385, *P* < 0.001), stenosis in the main lesion (OR = 1.247, 95% CI = 1.173–1.327, *P* < 0.001), DLO (OR = 1.034, 95% CI = 1.019–1.049, *P* < 0.001), DLD (OR = 1.061, 95% CI = 1.043–1.079, *P* < 0.001), and DLD/DLD (OR = 0.033, 95% CI = 0.010–0.117, *P* < 0.001) were the independent variables for predicting VA formation in MI patients.

**Conclusion:**

Our study first used quantified information of coronary lesions to establish a predictive model and proved that a longer DLD had the greatest potential in predicting the incidence of VA. Its related parameters including DLO and DLO/DLD ratio were also correlated with the incidence of VA. These findings may provide a new reference for the early identification of high-risk MI patients and preventing VA.

## Introduction

The short-term mortality of patients after myocardial infarction (MI) has substantially declined with advancements in percutaneous coronary intervention (PCI). However, the long-term prognosis of MI, which is affected by post-PCI complications, remains an impending clinical problem (1, 2). Ventricular aneurysm (VA) is a common complication of MI, with ~5–15% morbidity (3, 4). Because of the higher incidence of arrhythmias, thromboembolic phenomena, congestive heart failure, and cardiac rupture, the fatality of MI patients with VA is six times higher than that of patients without VA (4–6). It is believed that prophylactic treatment could prevent the development of VA (7, 8). Patients with total occlusion of the left anterior descending (LAD) artery, single-vessel disease, absence of previous angina, female sex, longer symptom-to-balloon time, and increased SYNTAX score are known to have a high risk of VA (9–13). However, the relationship between the specific coronary lesion parameters and VA formation has not been described in earlier studies. We hypothesized that these specific coronary lesion parameters are closely related to the severity of MI and the development of VA.

In this study, we retrospectively studied 52 MI patients with VA and 208 MI patients without VA as matched controls. The purpose of our study was to investigate the potential features of coronary angiography and to establish a more precise model for predicting VA formation.

## Methods

### Study Cohort

From June 2020 to July 2021, 52 patients with VA and previous emergency PCI for acute MI admitted to the Department of Cardiology of Tongji Hospital under Tongji University were retrospectively included in this study. The diagnosis of VA was reconfirmed by a clinician who underwent echocardiography according to the criteria of the Coronary Artery Surgery Study. For the control group, 208 MI patients without VA were retrospectively included and reviewed according to the records, Including age, female sex, BMI, blood pressure, heart rate, medical history, and drug intervention. This study was approved by the Ethics Committee of Tongji Hospital, Tongji University. Written informed consent was obtained from all patients regarding the analysis of clinical information for the purpose of scientific research.

### Coronary Angiography Parameters

Coronary angiograms were re-analyzed by two independent cardiologists who were blinded to the clinical data. The culprit lesion was defined as the site of acute coronary occlusion or the site of greatest narrowing with angiographically significant stenosis corresponding to electrocardiographic changes in non-occluded arteries. The curve length of the coronary artery was first measured in the form of pixels using ImageJ software and then converted to millimeters.

### Statistics Analysis

All experimental data were assessed using SPSS version 22.0 (IBM Corp, Armonk, NY). Data are presented as arithmetic means and standard deviations. Differences between groups were assessed using Student's *t*-test for continuous quantitative variables and χ^2^ test for qualitative variables. Independent factors were analyzed using conditional logistic regression analysis. The receiver operating characteristic (ROC) curve was used to determine the application value of continuous variables in predicting the formation of VA. Two-sided values with *P* < 0.05 were statistically significant.

## Results

### Baseline Characteristics

This study included 260 MI patients with and without VA (*n* = 52 and *n* = 208, respectively). The baseline characteristics of the patients are shown in Table 1. As shown in Table 1, there were no significant differences in age, female sex, BMI, blood pressure, heart rate, medical history, or drug intervention between the two groups of patients with MI. Compared to patients without VA, patients with VA had significant higher level of maximum serum cardiac troponin I (cTnI) (61.02 ± 23.71 vs. 46.51 ± 13.8, *P* < 0.001) and serum brain natriuretic peptide levels (1122.53 ± 484.59 vs. 967.73 ± 317.63, *P* = 0.034). Moreover, ST segment elevation was more frequent in patients with VA than in those without VA (67.3 vs. 44.2%, χ^2^ = 8.866, *P* = 0.003).

**Table 1:** Baseline characteristics compared between patients with and without VA.

	**Total**	**Non-VA patients**	**VA patients**	
*N*	260	208	52	
Age (years)	56.71 ± 11.57	56.19 ± 11.71	59.89 ± 11.23	*P* = 0.145
Female sex (%)	95(36.5%)	75(36.1%)	20(38.5%)	χ^2^ = 0.104 *P* = 0.747
BMI (kg/m^2^)	23.97 ± 2.21	23.84 ± 2.26	24.39 ± 1.78	*P* = 0.059
Systolic pressure (mmHg)	119.63 ± 17.47	119.46 ± 17.27	119.35 ± 17.98	*P* = 0.756
Diastolic pressure (mmHg)	60.71 ± 11.82	61.19 ± 11.98	58.24 ± 10.06	*P* = 0.192
Heart rate (bpm)	87.38 ± 18.57	86.94 ± 18.39	89.05 ± 19.42	*P* = 0.452
Smoke (%)	95(36.5%)	75(36.1%)	20(38.5%)	χ^2^ = 0.104 *P* = 0.747
**Blood test**				
PLT (×10^9^/L)	224.92 ± 43	226.92 ± 42.94	216.88 ± 42.28	*P* = 0.821
LDL-C (mmol/L)	4.46 ± 1.47	4.48 ± 1.46	4.38 ± 1.51	*P* = 0.68
cTnI max (ng/mL)	49.41 ± 17.27	46.51 ± 13.8	61.02 ± 23.71	*P* < 0.001
BNP (pg/mL)	998.69 ± 362.65	967.73 ± 317.63	1122.53 ± 484.59	*P* = 0.034
**Medical history**				
Hypertension (%)	115 (44.2%)	93 (44.7%)	22 (42.3%)	χ^2^ = 0.097 *P* = 0.755
Diabetes mellitus (%)	123 (47.3%)	98 (47.1%)	25 (48.61%)	χ^2^ = 0.015 *P* = 0.901
Hyperlipemia (%)	119 (45.8%)	95 (45.7%)	24 (46.2%)	χ^2^ = 0.004 *P* = 0.95
Atrial fibrillation (%)	22 (8.5%)	18 (8.7%)	4 (7.7%)	χ^2^ = 0.05 *P* = 0.824
**Drug intervention**				
Single antiplatelet (%)	146 (56.2%)	118 (56.7%)	28 (53.8%)	χ^2^ = 0.141 *P* = 0.708
Dual antiplatelet therapy (%)	114 (43.8%)	90 (43.3%)	24 (46.2%)	χ^2^ = 0.141 *P* = 0.708
Anticoagulative therapy (%)	22(8.5%)	18(8.7%)	4(7.7%)	χ^2^ = 0.05 *P* = 0.824

*VA, ventricular aneurysm; BMI, body mass index; PLT, platelet; LDL-C, low-density lipoprotein cholesterol; cTNI, maximum serum cardiac troponin I; BNP, brain natriuretic peptide*.

### Lesion Artery Characteristics

Table 2 presents the detailed lesion artery characteristics acquired using coronary angiography. Patients with VA had a significantly higher rate of single-vessel disease than those without VA (42.3 vs. 28.8%, *P* = 0.028). The most common culprit artery in patients with VA was the LAD (9.6 vs. 51.9% vs. 28.8 vs. 9.6%), and its proportion within the group was significantly higher than that in patients without VA (88.5 vs. 60.1%, χ^2^ = 12.821, *P* < 0.001). Moreover, the stenosis of main lesion in VA patients was also higher than those without VA (91.90 ± 5.08 vs. 82.90 ± 6.09, *P* < 0.001).

**Table 2:** Lesion artery characteristics of patients with and without VA.

	**Non-VA patients**	**VA patients**	
Single vessel disease (%)^a^	55(28.8%)	22(42.3%)	χ^2^ = 5.023 *P* = 0.028
Multi vessel disease (%)^a^	153(71.2%)	30(57.7%)	χ^2^ = 5.023 *P* = 0.028
Main lesion stenosis (%)^b^	82.90 ± 6.09	91.90 ± 5.08	*P* < 0.001
**Lesion artery**			
LM (%)	23(11.1%)	8(15.4%)	χ^2^ = 0.742 *P* = 0.389
LAD (%)	125(60.1%)	46(88.5%)	χ^2^ = 12.821 *P* < 0.001
LCX (%)	116(55.8%)	28(53.8%)	χ^2^ = 0.062 *P* = 0.876
RCA (%)	81(38.5%)	20(38.9%)	χ^2^ = 0.004 *P* = 1.000
**Lesion dimensions**			
DLO (mm)	56.07 ± 22.97	39.50 ± 16.10	*P* < 0.001
DLD (mm)	84.42 ± 23.72	116.12 ± 16.55	*P* < 0.001
DLO/DLD	0.77 ± 0.44	0.36 ± 0.17	*P* < 0.001

*LM, left main coronary artery; LAD, left anterior descent; LCX, left circumflex; RCA, right coronary artery; DLO, distance from lesion to ostia; DLD, distance from lesion to distal*.

a *Proportion of cases*.

b *Severity of main lesions*.

**Figure 1:**
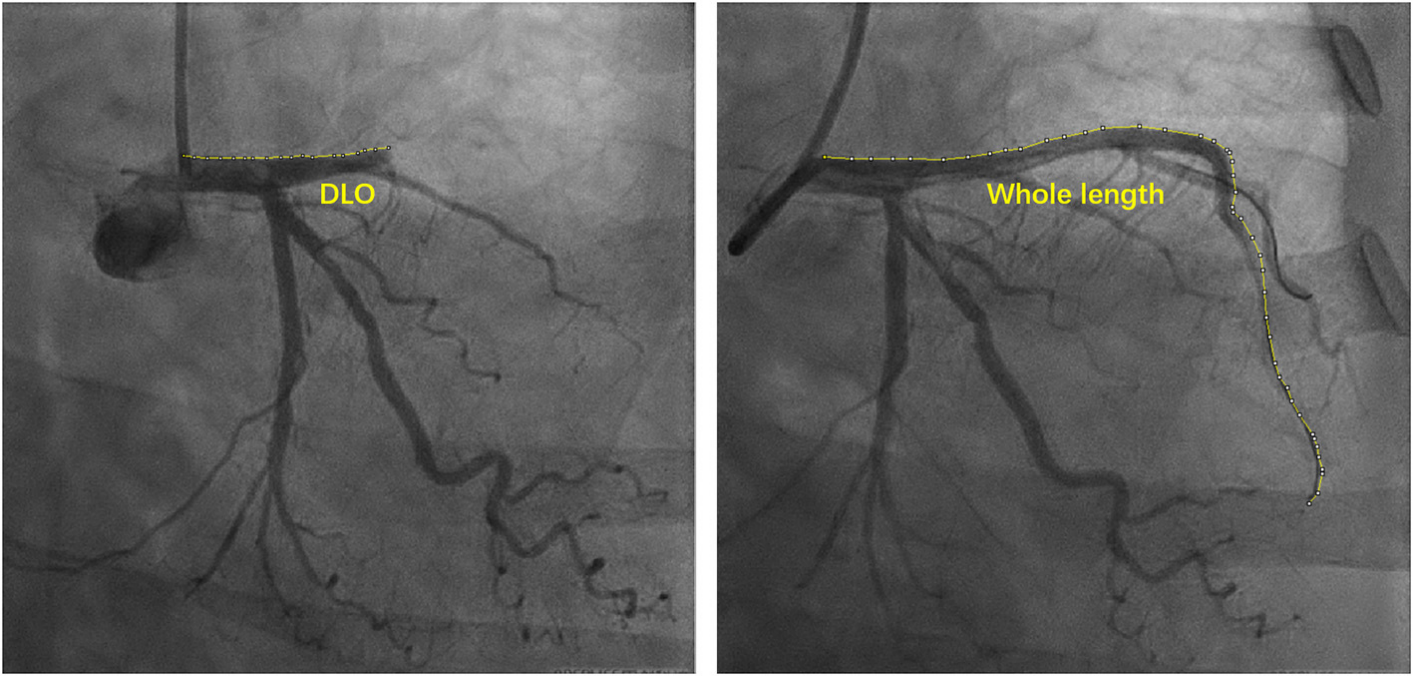
Schematic diagram of measuring curve length of coronary artery. DLO, distance from lesion to ostia.

The damage range of the myocardium during MI is believed to be the main factor in VA formation (14). As shown in Figure 1, we measured the curve length of the distance from the main culprit lesion to its ostia (DLO) and to its distal (DLD) to estimate the damage range due to the main lesion. From our observations, the DLO curve length was significantly shorter in VA cases than in patients without VA (39.50 ± 16.10 vs. 56.07 ± 22.97, *P* < 0.001), whereas the DLD curve length of VA patients was much longer than that of patients without VA (116.12 ± 16.55 vs. 84.42 ± 23.73). The DLO/DLD ratio was also significantly lower in patients with VA than in patients without VA (0.36 ± 0.17 vs. 0.77 ± 0.44, *P* < 0.001).

### Logistic Regression and Related Factors of VA

The binary logistic regression analysis revealed elevated ST segment (odds ratio [OR] = 0.385, 95% confidence interval [CI] = 0.203–0.731, *P* = 0.004), maximum cTNI (OR = 1.046, 95% CI = 10.27–1.066, *P* < 0.001), serum brain natriuretic peptide (BNP) (OR = 1.001, 95% CI = 1.000–1.002, *P* = 0.007), LAD as culprit artery (OR = 5.091, 95% CI = 2.080–12.457, *P* < 0.001), single-vessel disease (OR = 1.809, 95% CI = 0.967–3.385, *P* < 0.001), main lesion stenosis (OR = 1.247, 95% CI = 1.173–1.327, *P* < 0.001), DLO (OR = 1.034, 95% CI = 1.019–1.049, *P* < 0.001), DLD (OR = 1.061, 95% CI = 1.043–1.079, *P* < 0.001), and DLD/DLD (OR = 0.033, 95% CI = 0.010–0.117, *P* < 0.001) as the independent predictors for VA formation in MI patients (Table 3).

**Table 3:** Binary logistic regression analysis of factors related to the formation of VA.

	**Odds Ratio**	**95% CI**	
cTnI max	1.046	1.027–1.066	*P* < 0.001
BNP	1.001	1.000–1.002	*P* = 0.007
LAD	5.091	2.080–12.457	*P* < 0.001
Single vessel disease	1.809	0.967–3.385	*P* < 0.001
Main lesion stenosis	1.247	1.173–1.327	*P* < 0.001
DLO	1.034	1.019–1.049	*P* < 0.001
DLD	1.061	1.043–1.079	*P* < 0.001
DLO/DLD	0.035	0.010–0.117	*P* < 0.001

*cTnI max, max cardiac troponin I; BNP, brain natriuretic peptide; LAD, left anterior descending; RCA, right coronary artery; DLO, distance from lesion to ostia; DLD, distance from lesion to distal*.

### ROC Curve Analysis

**Figure 2:**
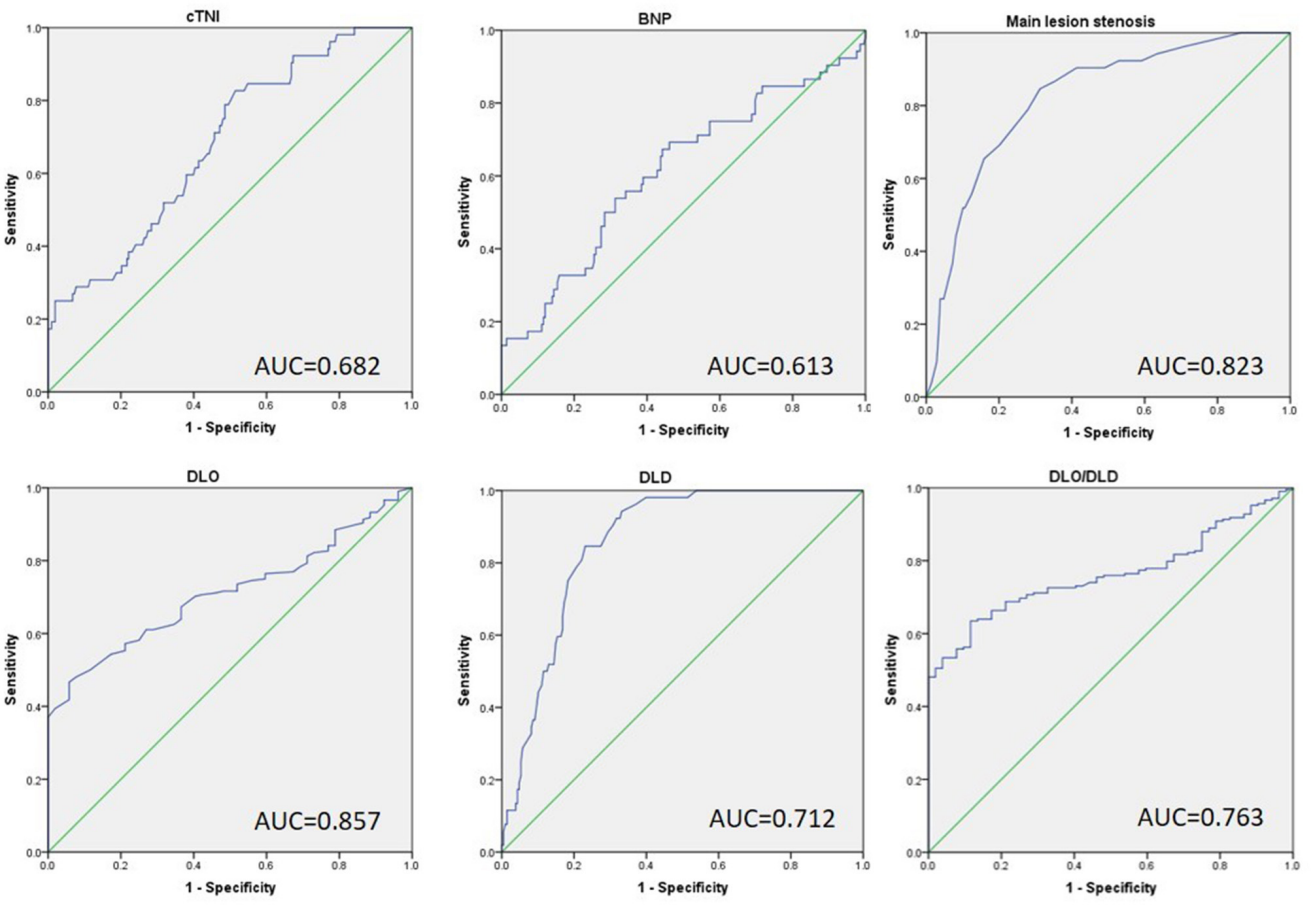
The receiver operative characteristic curve of independent factors predicts the development of VA in patients with previous MI. cTNI max, max cardiac troponin I; BNP, brain natriuretic peptide; DLO, distance from lesion to ostia; DLD, distance from lesion to distal.

To determine the ideal strain cutoff values of these independent variables and establish a reliable model for predicting VA formation in MI patients, continuous variables from the logistic regression results were analyzed using the ROC curve. As shown in Figure 2 and Table 4, cTnI ≥ 45.05 was identified as the optimal cutoff to predict VA formation with an area under the curve (AUC) of 0.682, providing a sensitivity of 82.7% and a specificity of 48.6%. Serum BNP ≥ 956.25 had 67.3% sensitivity, 55.8% specificity, and an AUC of 0.613. A main lesion stenosis of ≥ 85.5 has 90.4% sensitivity, 68.7% specificity, and an AUC of 0.855. DLO ≤ 58.5 had 52.9% sensitivity, 86.5% specificity, and an AUC of 0.724, while DLD ≥ 92.5 had 96.2% sensitivity, 66.8% specificity, and an AUC of 0.866. A DLO/DLD ratio of ≤ 0.581 had 63.0% sensitivity, 90.4% specificity, and an AUC of 0.774.

**Table 4:** ROC analysis.

	**Cut-off value**	**AUC**	**Sensitivity (%)**	**Specificity (%)**	
cTnI max	45.05	0.682	82.7	48.6	*P* < 0.001
BNP	956.25	0.613	67.3	55.8	*P* = 0.012
Main lesion stenosis	85.5	0.863	90.4	68.7	*P* < 0.001
DLO	58.5	0.724	52.9	86.5	*P* < 0.001
DLD	92.5	0.866	96.2	66.8	*P* < 0.001
DLO/DLD	0.581	0.774	63.0	90.4	*P* < 0.001

*cTnI max, max cardiac troponin I; BNP, brain natriuretic peptide; DLO, distance from lesion to ostia; DLD, distance from lesion to distal*.

## Discussion

Research on the pathogenesis of ventricular aneurysms has gradually improved in the last 30 years (14, 15). Currently, VA is considered to originate from weakened myocardial tissue at the infarct site, which bulges outward under the pressure of the ventricular cavity. After MI, necrotic myocardial tissue is replaced by fibrotic tissue, which has no contractile capacity. The contractile function of the surviving myocardium around the fibrotic tissue is overcompensated and causes a reverse interaction that finally thins the ventricular wall, which leads to the formation of VAs. The natural prognosis of VA is dismal, with a 10-year survival rate of only 60% in untreated patients (16–19). Complications resulting from VA, such as heart failure, cardiac rupture, ventricular thrombus, and malignant arrhythmia, severely influence the quality-of-life of patients. However, most patients with VA have no symptoms in the early stage and can only be detected radiographically by echocardiography a few weeks or even months after the onset of disease (20, 21).

Presently, some epidemiological studies have preliminarily screened out the risk factors for VAs after MI, yet there are great differences among them. A case-control study including 193 patients showed that abnormalities in the glomerular filtration rate and serum ferritin level were independent risk factors for VA after AMI (22). Tikiz et al. screened four independent risk factors (single-vessel disease, previous history of angina pectoris, total occlusion of LAD, and female sex) (9). The retrospective analysis by Zhang et al. considered age, onset-to-treatment time, anterior wall infarction, leukocyte count, and left ventricular ejection fraction as the predictors of VA after AMI to a certain extent (3). A recent study focused on the time from onset to the beginning of treatment and found that symptom-to-balloon time and SYNTAX score (Synergy between PCI with TAXUS drug-eluting stent and Cardiac Surgery) could be reliable risk factors for the occurrence of VA (11). The above studies preliminarily identified some independent risk factors of VA after MI by logistic regression analysis based on the baseline characteristics, laboratory examination, and rough analysis of criminal blood vessels of the patients with previous MIs. However, those models still had room for improvement regarding the sensitivity and specificity of their predictive capability.

Baseline data of our cohort showed significant differences in the maximum serum cTNI levels and serum BNP levels between patients with VA and those without VA, which was consistent with previous reports. Likewise, the angiographic analysis showed that patients with VA had a higher incidence of LAD as a lesion artery, single-vessel disease, and more severe stenosis in the main lesion. In contrast to previous studies, however, door-to-balloon time in our study was not different between the two groups, which suggested that some MI patients still have a risk for VA underling the need for undergoing complete revascularization within appropriate time. In most previous studies, the sites of coronary artery lesions were described with blurred position information, including proximal obstruction and diffuse mid or distal stenosis. To our knowledge, our study is the first to use quantified information of coronary artery lesions to establish a predictive model and correlate it with the incidence of VA. We found that the curve length of the distance from the main culprit lesion to its ostia or distal ostia independently predicted the occurrence of VA. The DLO/DLD ratio indirectly reflecting the cardiac infarct size in MI patients additionally shows a strong correlation with VA. Our study provides a new reference for the early identification of high-risk MI patients and the prevention of VA.

### Limitations

The study has some limitations. First, this was a single-center retrospective analysis with a small sample size. Limited to some study conditions, we adopted a retrospective case-control study for ensuring that the study could include as many cases as possible. At the same time, case-control study could save both time cost and economic cost. A multicenter prospective study with a larger sample size should be conducted to verify our inferences. Second, using the traditional manual method to analyze specific parameters in the coronary angiographic images is relatively subjective and unstable. Automatic recognition and measurements based on artificial intelligence algorithms should be further developed in the future.

## Conclusion

In this study, we first used quantified information of coronary lesions to establish a predictive model and proved that a shorter DLO, longer DLD, and decreased DLO/DLD ratio were independently correlated with the incidence of VA. DLD was with highest potential to be a predictor. These findings may provide a new reference for the early identification of high-risk MI patients and preventing VA.

## Data Availability Statement

The raw data supporting the conclusions of this article will be made available by the authors, without undue reservation.

## Ethics Statement

The studies involving human participants were reviewed and approved by Ethics Committee of Tongji Hospital Affiliated to Tongji University. The patients/participants provided their written informed consent to participate in this study. Written informed consent was obtained from the individual(s) for the publication of any potentially identifiable images or data included in this article.

## Author Contributions

PY collected the clinical information of the enrolled patients, analyzed the data and drafted the manuscript. PX and YT collected the clinical information of the enrolled patients and organized the data. JX and YL supervised the research design and proofreading the manuscript. All authors contributed to the article and approved the submitted version.

## Funding

This work was supported by the grants from the National Natural Science Foundation of China (No. 82070411 to JX) and Scientific Research Project of Shanghai Municipal Health Commission (No. 202040077 to YL).

## Conflict of Interest

The authors declare that the research was conducted in the absence of any commercial or financial relationships that could be construed as a potential conflict of interest.

## Publisher's Note

All claims expressed in this article are solely those of the authors and do not necessarily represent those of their affiliated organizations, or those of the publisher, the editors and the reviewers. Any product that may be evaluated in this article, or claim that may be made by its manufacturer, is not guaranteed or endorsed by the publisher.

## Abbreviations

AUC: area under the curve
AMI: Acute myocardial infarction
BMI: body mass index
BNP: brain natriuretic peptide
cTNI max: max cardiac troponin I
DLO: distance from lesion to ostia
DLD: distance from lesion to distal
LDL-C: low-density lipoprotein cholesterol
LM: left main coronary artery
LAD: left anterior descent
LCX: left circumflex
RCA: right coronary artery
ROC: receiver operating characteristic
PCI: percutaneous coronary intervention
VA: ventricular aneurysm.
